# Differential Effects of Drinking Water Quality on Phagocyte Responses of Broiler Chickens Against Fungal and Bacterial Challenges

**DOI:** 10.3389/fimmu.2020.00584

**Published:** 2020-04-07

**Authors:** Juan A. More-Bayona, Débora Torrealba, Caitlin Thomson, Jeremy Wakaruk, Daniel R. Barreda

**Affiliations:** ^1^Laboratory of Immunology and Animal Health, Department of Biological Sciences, University of Alberta, Edmonton, AB, Canada; ^2^Department of Agricultural, Food and Nutritional Sciences, University of Alberta, Edmonton, AB, Canada

**Keywords:** drinking water quality, phagocyte function, acute inflammation, fungal and bacterial challenges, comparative immunology

## Abstract

Combinatorial effects of xenobiotics in water on health may occur even at levels within current acceptable guidelines for individual chemicals. Herein, we took advantage of the sensitivity of the immune system and an avian animal model to examine the impact of xenobiotic mixtures on animal health. Water was derived from an underground well in Alberta, Canada and met guidelines for consumption, but contained a number of contaminants. Changes to chicken immunity were evaluated following acute (7d) exposure to contaminated water under basal and immune challenged conditions. An increase in resident macrophages and a decrease in CD8+ lymphocytes were identified in the abdominal cavity, which served as a relevant site where immune leukocytes could be examined. Subsequent intra-abdominal immune stimulation detected differential *in vivo* acute inflammatory responses to fungal and bacterial challenges. Leukocyte recruitment into the challenge site and activation of phagocyte antimicrobial responses were affected. These functional responses paralleled molecular changes in the expression for pro-inflammatory and regulatory genes. In all, this study primarily highlights dysregulation of phagocyte responses following acute (7d) exposure of poultry to contaminated water. Given that production food animals hold a unique position at the interface of animal, environmental and human health, this emphasizes the need to consider the impact of xenobiotic mixtures in our assessments of water quality.

## Introduction

Water is the most important element for any living organism (1, 2), essential to immune function and the maintenance of homeostasis. This translates into meaningful implications for animal health and performance. To date, most studies on water contamination have focused on the effects of individual contaminants, and concentration values that exceed recommended levels. Given the abundance of different contaminants in the environment, we and others believe that added emphasis should be placed on the combinatorial biological effects of chemicals in these mixtures and their by-products (3–6). This, however, requires a focus on functional read-outs rather than conventional examination for the presence of a growing list of individual contaminants using chemical analyses. Further, it requires added availability of reagents to capture the impact of these mixtures on a range of terrestrial and aquatic organisms.

Production food animals hold a unique position at the interface of animal, environmental and human health (7–9). Among others, they serve as important sentinels for pathogens and xenobiotics. In the present study, we identified multiple contaminants present in underground water and assessed their combinatorial effects on chicken immunity following acute 7-day exposure. We focused on phagocyte responses, first measuring pre-challenge numbers for resident macrophages and their activation state, as indicators of changes to basal immunity. We then paired these with evaluation of molecular and cellular responses following *in vivo* intra-abdominal challenges with two well-defined fungal and bacterial models. Our fungal model, zymosan, has been widely used in comparative systems and has provided important insights into mechanisms governing the induction and control of acute inflammation (10–15). Our bacterial model, Salmonella enterica serovar Typhimurium (ST), is among the most common and relevant enteric pathogens for the food production industry and public health (16–18). ST is well established to engage phagocyte subsets, inducing marked heterophil and macrophage recruitment and activation in infected chickens (17, 19). Evaluation of these early changes in phagocyte numbers and function showed that contaminants in underground raw water, even within acceptable concentrations, induce marked effects on bird immunity.

## Materials and Methods

### Chickens

Three-week-old Ross 708 broiler chickens (*Gallus gallus*) were used. All animals were housed in the Poultry Research Facility of the Agriculture, Food and Nutritional Sciences at the University of Alberta. Animals were grouped into two experimental treatments: raw underground well water (raw well water) and tap water control group (tap water). Animals were maintained according to guidelines specified by the Canadian Council on Animal Care, and protocols approved by the University of Alberta Animal Care and Use Committee. Maximum efforts were made to minimize animal stress and chickens terminated by cervical dislocation and exsanguination.

### Treatment

Drinking water was administrated for 7 days. Following water treatment, chickens were abdominally challenged using zymosan (2.5 mg, Sigma Aldrich), resuspended in 500 μl of 1x PBS^−/−^ (no calcium, no magnesium). Zymosan is a well-established pathogen mimic obtained from *Saccharomyces cerevisae*, which promotes immune cell activation and function through mannose and β-glucans receptors. Previous *in vitro* and *in vivo* studies have shown that zymosan phagocytosis results in activation of pro-inflammatory responses that include induction of pro-inflammatory cytokines, production of reactive oxygen and nitrogen intermediates and increased infiltration of leukocytes, predominantly neutrophils (20–26). The zymosan dose was selected because it allowed for natural transition to resolution of acute inflammatory processes, thus providing a self-resolving peritonitis model for *in vivo* examination of the impact of water quality on bird immunity (10, 27–30). For bacterial challenges, we focused on a *Salmonella enterica* serovar typhimurium X4232 strain (ST). This is a nalidixic-acid resistant strain that have been broadly used to examine inflammation in multiple animal models. In our experimental design, ST was cultured on xylose lysine deoxycolate agar (XLD) for 24 hours at 37°C followed by culture in LB broth at 37°C at 150 rpm for 20 h to obtain 10^9^ CFU/ml of culture broth. ST was heat-killed at 80°C in a water bath for 1 h an d resuspended in 1x PBS^−/−^. The goal was to provide a bacterially induced self-resolving immune challenge that would not suffer from confounding factors associated with microbial growth. Heat-killed ST (HKST; 10^9^ CFU) was resuspended in 500 μl and keep at −4°C until injection.

### Abdominal Lavage

Chickens were euthanized via cervical dislocation and animals were bled to minimize potential blood contamination into the abdominal cavity, as previously described (13, 14). Leukocytes were recovered by injecting 20 ml of incomplete RPMI media into the lower left quadrant at the abdominal site. Harvested leukocytes were kept at 4°C. Non-injected chickens were used as negative controls.

### Definition of Phagocyte Populations

Phagocytes were identified using imaging flow cytometry along with modified Wright-Giemsa staining (Hema3). For Hema3 (Fischer Scientific), leukocyte cytospins were stained according to the manufacturer's specifications and analyzed through light microscopy. For imaging flow cytometry, a dot plot of events using area (size, x-axis) vs intensity channel 6 (internal complexity, y-axis) was created to define the different subpopulations using leukocyte morphology and nuclear staining (Draq5). Monoclonal antibodies further helped to define specific leukocyte subpopulations. Monocyte/macrophage labeling was performed using the KUL01 antibody marker (Abcam). This antibody recognizes a homolog of the mammalian mannose receptor C-type, MRC1 (also called CD206) (31, 32). The KUL01 antibody was added to a final concentration of 1:1000, followed by 30 min incubation at 41°C. Cells were washed in PBS^−/−^ and fixed in 1% of formaldehyde. PE anti-chicken CD4^+^ and Cy5 anti-chicken CD8^+^ (Abcam) were used for CD4^+^ and CD8^+^ T lymphocytes staining, respectively. Anti CD4^+^ antibody was added at 1:1000 dilution and anti CD8^+^ T lymphocytes antibody at 1:2000 dilution. Leukocytes were incubated at −4°C for 30 min and followed by 20 min at room temperature. Hoechst 33342 was added as nuclear staining.

### Analysis of Phagocyte Function

ROS production was measured using CellROX (Molecular probes), as previously described (13, 14). NO production was determined using DAF-FM diacetate (Molecular Probes) oxidation staining. DAF-FM diacetate reagent was diluted 1:25 in 1x PBS^−/−^ and 4 μl was added for incubation for 30 min at 41°C. Phagocytes were washed with 1x PBS^−/−^ and fixed in 1% formaldehyde.

### Gene Expression

Abdominal leukocytes were kept in Trizol (Thermo Fisher Scientific), stored in liquid nitrogen and total RNA was extracted following manufacturer's specifications. RNA concentration and quality were evaluated using Nanodrop ND-1000 (Thermo Fisher Scientific) and Bioanalyser-2100 with the RNA 6000 Nano Kit (Agilent Technologies), respectively. Samples had a RIN higher than 7.5. cDNA was synthesized with 650 ng of total RNA in a final volume of 20 μl using iScript Kit (BioRad). Transcripts of r28s and ACTB were used as reference genes for quantification purposes. qPCR was carried out using SYBR Green (prepared by Molecular Biology Services Unit staff at the University of Alberta), 500 nM of primers and 2.5 μl of cDNA previously diluted in 10 μl of final volume; and evaluated using the QuantStudio 6 Flex Real-Time PCR System (Applied Biosystems). Primers for qPCR are described in Table 1. Relative quantification was performed according to Livak's method (33). Samples were run in triplicates and results were statistically analyzed using Two-way ANOVA followed by Sidak's multiple comparison test to estimate differences between treatments.

**Table 1:** Primers used for qPCR analysis.

**Gene**	**Primer name**	**Sense**	**Sequence**	**Accession number**
**Interleukin 2**	**IL-2**	Fw	ACCGGAAGTGAATGCAAGAT	AF000631
		Rv	AGTGGTCCCAGAATGGACAG	AF000631
**Interleukin 8**	**IL-8**	Fw	GGCTTGCTAGGGGAAATGA	AJ009800
		Rv	AGCTGACTCTGACTAGGAAACTGT	AJ009800
**Tumoral necrosis factor alpha**	**TNF-α**	Fw	GTTGACTTGGCTGTCGTGTG	AY765397.1
		Rv	TCAGAGCATCAACGCAAAAG	AY765397.1
**Interleukin 1 beta**	**IL-1β**	Fw	AGGTCAACATCGCCACCTAC	NM_204524.1
		Rv	ACGAGATGGAAACCAGCAAC	NM_204524.1
**Transforming growth factor beta**	**TGF-β**	Fw	CGACCTCGACACCGACTACT	NM_001318456.1
		Rv	CCACTTCCACTGCAGATCCT	NM_001318456.1
**Inducible nitric oxide synthase**	**i-NOS**	Fw	CTCTCACAGGCCTTGACATATT	D85422.1
		Rv	CAGTCTCTGTTTGTCTCCTTCC	D85422.1
**Ribosomal 28 subunit**	**r28S**	Fw	GGCGAAGCCAGAGGAAACT	FM165415
		Rv	GACGACCGATTTGCACGTC	FM165415
**Actin beta**	**ACTB**	Fw	CCAGACATCAGGGTGTGATGG	AJ719605
		Rv	CTCCATATCATCCCAGTTGGTGA	AJ719605

### Statistical Analysis

GraphPad Prism software was used to assess statistical differences and significance between groups using two-way ANOVA and Sidak's multiple comparison tests. Statistics with *p* < 0.05 were considered significant.

## Results

### Raw Water Source Selection and Analysis

Raw well water was obtained from a poultry producer in south eastern Alberta, Canada located in an area of high chemical and high fertilizer expense (20, 34), and in close proximity to point source contamination from a natural gas extraction well (located approximately 500 m from water source). The local producer uses this well water as the water source for one chicken coup. Unique to this producer is another nearby coup under his operation, which uses municipal tap water as its water source. We considered this an excellent opportunity to examine source effects as both cohorts were managed by the same producer using equivalent procedures, with only the water being a variable. There were also anecdotal accounts of animal health and performance effects, and the producer had noted a significant difference in the quality of litter across both locations. The litter of the coup where well water is consumed was said to contain higher levels of moisture likely due to a laxative effect. This required the facility have greater ventilation to maintain suitable humidity.

Water was analyzed according to CALA (Canadian Association for Laboratory Accreditation) and ISO17025 standards. Parameters selected for analysis are known to have detrimental effects on human and animal health (21). Higher levels of ammonia, phosphates or potassium for the area pointed to a presence of contaminants stemming from agricultural activities. A summary of the results found in the raw water source used and comparisons to tap water are provided in Table 2. From more than 150 elements tested in the well water used, some were found to be near but above acceptable guidelines, including calcium carbonate and bicarbonate, sulfates and sodium. We also found trace concentrations of total penta chloro-dibenzo-p-dioxin and octa chloro-dibenzo-p-dioxin, members of a group known as dioxins. Additional parameters were significantly lower than maximum acceptable levels and, thus, not included in the table.

**Table 2:** Summary of xenobiotics found in raw and tap water.

**Parameter name**	**Units**	**Results** **(raw)**	**Results** **(tap)**	**Max. acceptable concentration**
Total dissolved solids	ppm	970	230	1000
Chloride	ppm	23	6.5	200
Ph	pH	8.40	8.03	7.0–10.5
Alkalinity (CaCO3, Bicarb)	ppm	570	170	300
Nitrates	ppm	<0.01	0.32	25
Sulfates	ppm	220	77	200
Iron	ppm	0.06	<0.06	0.3
Calcium	ppm	6.6	46	60
Copper	ppm	0.0034	0.42	0.6
Magnesium	ppm	0.93	13	125
Manganese	ppm	0.015	<0.0040	0.05
Sodium	ppm	390	17	150
Zinc	ppm	0.0061	0.034	1.5
Total Penta CCD^*^	pg/L	1.54	N/A	2^**^-2.3^***^pg/Kg/day
Octa CDD^*^	pg/L	5.7	N/A	2^**^-2.3^***^pg/Kg/day
Fecal coliforms	CFU	0	0	0

* chloro-dibenzo-p-dioxin.

** World health organization (35).

*** *European Commisision's Scientific Committee on Food (36)*.

### Raw Well Water Increases Basal Numbers of Abdominal Resident Macrophages While Decreasing CD8^+^ T Lymphocytes in Chickens

**Figure 1:**
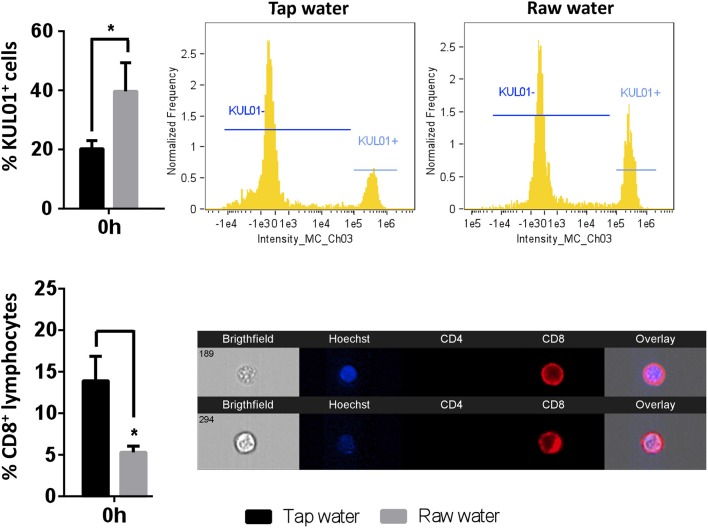
Short-term exposure to raw well drinking water impacts resident macrophage and lymphocyte numbers in broiler chickens. Birds were exposed to well water as drinking source for 7d. Leukocytes were isolated through abdominal lavage and incubated with anti-PE-KUL01 antibody, to determine the proportions of resident macrophages. CD8 T lymphocytes were identified using a Cy5 anti-chicken CD8 antibody. Data represents mean ± SEM (*n* = 6). Differences were assessed using two-way ANOVA and Sidak's multiple comparison test. *p < 0.05.

We first evaluated whether acute exposure to raw well water resulted in changes to basal chicken immunity and used the abdominal cavity as a relevant site where immune leukocytes could be examined. We identified a higher proportion of abdominal macrophages in animals that consumed raw well water for 7d compared to animals which were provided with normal tap water, even though the former still met stringent Canadian drinking water guidelines for consumption (Figure 1, *p* = 0.012). In contrast, we observed a reduced proportion of CD8+ T lymphocytes in the raw well water group compared to the tap water control group (Figure 1, *p* = 0.023). No changes were observed in the proportions of CD4+ leukocytes between control and raw well water exposed groups under non-immune challenged conditions (data not shown).

### Drinking Water Quality Affects Leukocyte Recruitment During Acute Inflammation

**Figure 2:**
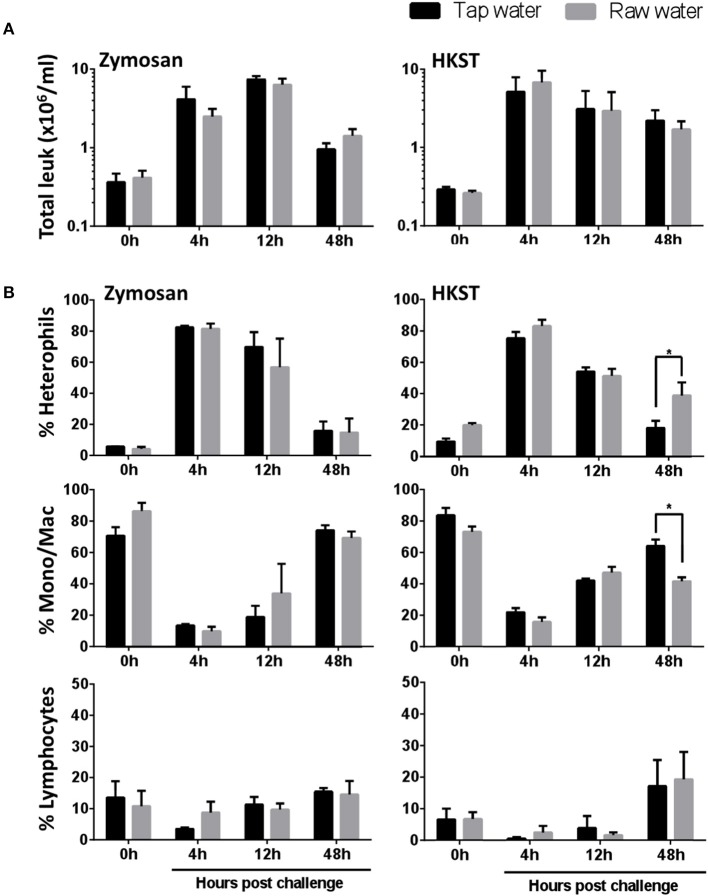
Raw well water differentially affects leukocyte recruitment following fungal and bacterial *in vivo* stimulation. Following 7d raw well water consumption, birds were challenged *in vivo* via intra-abdominal route using zymosan (fungal) or heat killed Salmonella enterica serovar Typhimurium (HKST; bacterial). Abdominal lavages were performed at 0, 4, 12, and 48 h post intra-abdominal challenge. **(A)** Total leukocytes were counted using a hemocytometer and light microscopy. **(B)** The proportions of heterophils, monocyte/macrophages and lymphocytes were determined using Imaging flow cytometry along with conventional Wright Giemsa staining. Data represents mean ± SEM (*n* = 7). Differences were evaluated using two-way ANOVA and Sidak's multiple comparison test. *p < 0.05.

Results above showed that drinking water quality affected resident leukocyte numbers even when exposure was limited to 7 days. We then evaluated the impact under immune challenged conditions. Birds were injected intra-abdominally with zymosan or heat killed Salmonella enterica serovar Typhimurium (HKST) following this same 7d water exposure. Leukocyte recruitment was assessed 4, 12 and 48 h after *in vivo* challenge (Figure 2A). Values were not significantly different across raw well water and tap water (control) treatments when total leukocyte migration numbers were considered. Higher resolution analyses, however, showed extended heterophil retention within the abdominal cavity for birds in the raw well water group following *in vivo* bacterial challenge (Figure 2B, 48 h post-challenge, *p* = 0.019). Concurrently, the proportion of monocyte/macrophages remained lower in the raw well water group at this 48 h time point (Figure 2B, *p* = 0.004). Although our fungal challenge showed similar kinetics of leukocyte recruitment, dominated by heterophil infiltration, the effect on heterophil retention was limited to the bacterial challenge (Figure 2B).

### Drinking Water Quality Affects Phagocyte Function at the Immune Challenge Site

**Figure 3:**
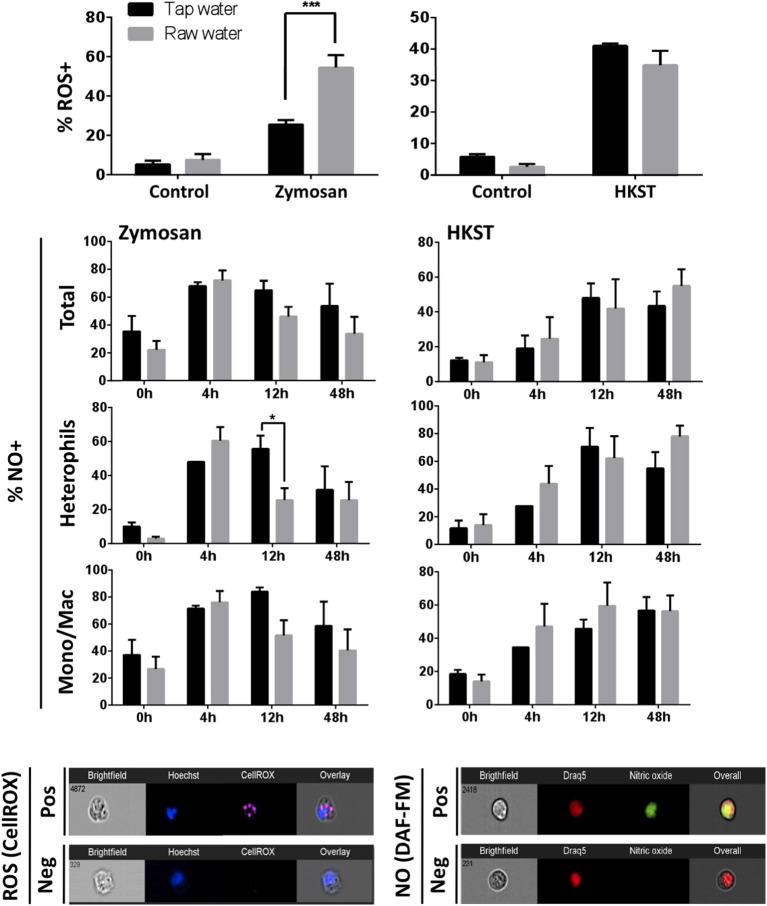
Raw well water treatment differentially impacts phagocyte antimicrobial responses. Birds were exposed to well water as drinking source for 7d and subsequently exposed to fungal or bacterial *in vivo* stimulation. Respiratory burst (ROS production) capacity in isolated leukocytes was assessed using CellROX. Nitric oxide (NO) production was determined using DAF-FM diacetate. Data represents mean ± SEM (*n* = 5). Significant differences were identified using two-way ANOVA and Sidak's multiple comparison test (***p < 0.001; *p < 0.05). Representative images from an ImageStream MKII flow cytometer show positive (pos) and negative (neg) cells following staining with CellROX and DAF-FM diacetate.

ROS and NO production were selected as highly conserved antimicrobial mechanisms of immune defense. Fungal stimulation led to a higher proportion of ROS producing leukocytes in chickens that consumed raw well water (60% compared to 30% ROS producing leukocytes in control tap water group; Figure 3, *p*= 0.0009). In contrast, no difference was observed between tap (control) and raw well water groups following heat-killed Salmonella stimulation. Chicken NO production was also affected by drinking water quality. However, unlike ROS this was not evident when total leukocyte population was considered. Higher resolution analysis at the single cell level using imaging flow cytometry showed that levels of heterophil NO production were downregulated by 12 h post zymosan challenge in animals exposed to raw well water, compared to the control group which remained high at this time point (Figure 3, *p* = 0.041). As with production of reactive oxygen intermediates, the impact on NO production was limited to our fungal challenge.

### Raw Well Water Promoted Expression of Pro-inflammatory Leukocyte Genes During Acute Inflammation

**Figure 4:**
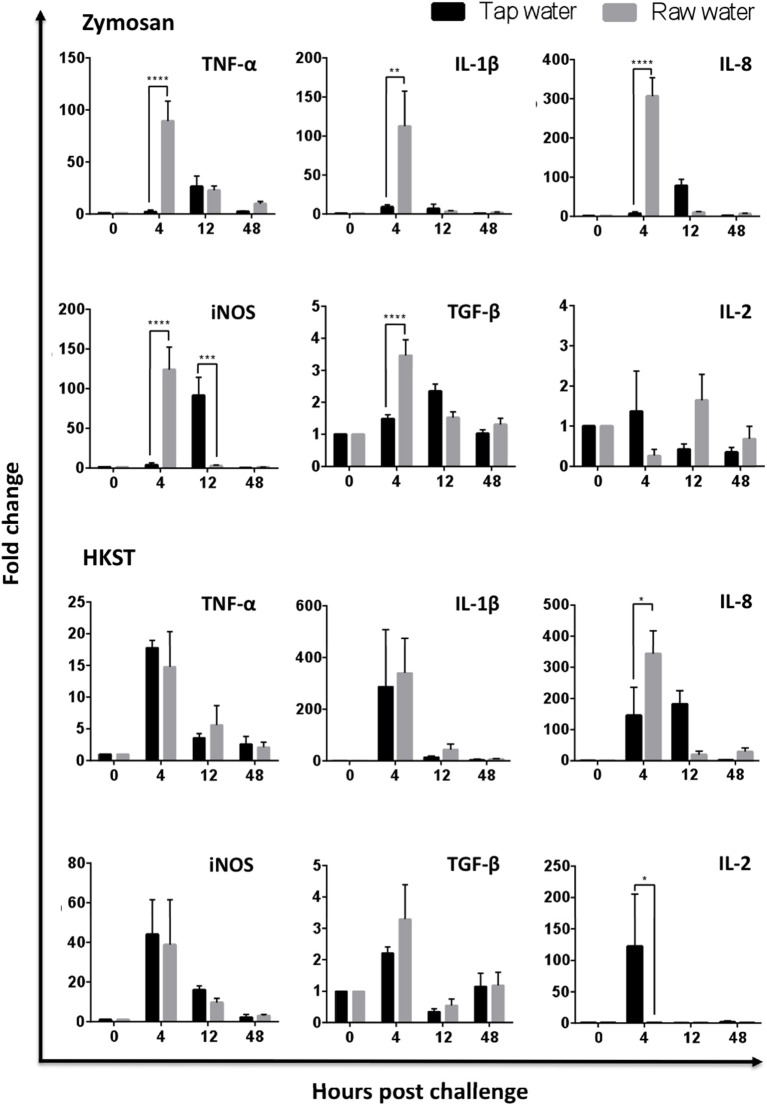
Raw well water treatment induces changes in leukocyte expression of pro-inflammatory and regulatory genes. Birds were exposed to well water as drinking source for 7d and subsequently exposed to fungal or bacterial *in vivo* stimulation. Abdominal leukocytes were harvested at 0, 4, 12, and 48 h post challenge. Gene expression was analyzed by qPCR. Data represents mean ± SEM (*n* = 4). Significant differences were analyzed using two-way ANOVA and Sidak's multiple comparison test. *p < 0.05, **p < 0.01, ***p < 0.001, ****p < 0.0001.

The impact of raw well water on leukocyte recruitment and function highlighted above paralleled changes observed at the molecular level. Effects were most pronounced in the zymosan-challenged group and impacted both kinetics and absolute levels of pro-inflammatory gene expression. Differences were evident as early as (4 h), where leukocytes derived from birds supplied with raw well water showed significantly higher levels of gene expression for TNFα, IL-1β, IL-8, and iNOS (Figure 4; TNFα *p* < 0.001; IL-1β *p* = 0.0013; IL-8 *p* < 0.0001; iNOS *p* < 0.0001). Notably, anti-inflammatory cytokine gene expression (TGF-β) was also upregulated early in the acute inflammatory response among leukocytes derived from the raw well water group, consistent with dysregulation of the immune response (Figure 4, *p* < 0.0001). Parallel experiments using our heat killed Salmonella enterica serovar Typhimurium *in vivo* challenge also displayed a dysregulated phenotype with upregulation of IL-8 and downregulation of IL-2 in the raw well water group. However, the effect was less marked than with the fungal *in vivo* challenge model shown above.

## Discussion

Assessment of water quality has become more challenging given the number and variety of xenobiotics that can enter the water supply from various sources. For pesticides and fertilizers, monitoring studies have shown that they are reaching Canada's water resources (22–25). While concentrations of these chemicals in water are generally low, they are commonly detected, particularly in regions of significant urban or agricultural development. Overall, farmland applications of pesticides and fertilizers have almost tripled in Alberta over the last 25 years (23). For pesticides, Alberta shows the second-highest amount of pesticide utilization in Canada, and although the relatively dry climate reduces the potential risk for water contamination throughout the year, significant risk still exists during episodes of surface water runoff (26). Unfortunately, the River Pesticide Index from which these values are derived from does not measure the risk to aquatic life, irrigated crop production, or drinking water sources (25). At the same time that agriculture operations continue to expand in this province, both the number and size of smaller communities near agricultural centers in the North and South Saskatchewan River Basins continue to increase (37, 38). These smaller communities display the highest vulnerability to water contamination episodes because of the proximity to the sources of contamination, their reliance of the water resources for drinking water, recreation, and irrigation of field crops, and because personnel and infrastructure for water treatment is often limited compared to larger population centers (39–41). As such, the convergence between expanding agricultural operations and local rural populations creates added risk for occurrence of acute and chronic diseases associated with exposure to pathogens and/or chemicals. In one example, the high levels of mixed animal agriculture in the Oldman river region have already been linked to one of the highest incidences of gastroenteritis in Canada (42, 43). These issues are not limited to Canada, but increasingly relevant globally (44–48).

Production animals fill a central position at the interface environment, animal and human health. They serve as sentinels for multiple infections and environmental contaminants, and impacts to their health status can facilitate the spread of infectious agents to consumers and the environment. Given the broad use of water resources for production animal rearing both geographically and throughout an animal's development, drinking water can play a significant and sustained role in the health of these animals. In this study, we identified no instances of morbidity or mortality in any of the treatment groups. However, we identified multiple effects of drinking water on bird immunity, even though this well water met stringent Canadian drinking water guidelines for consumption. Short-term (7d) exposure to xenobiotic mixtures through drinking water changed the resident leukocyte profile of test birds, with greater numbers of macrophages and lower numbers of CD8+ lymphocytes in the chicken abdominal cavity. We expect that these basal changes are associated with changes in the capacity of these birds to recognize and respond to pathogen infiltration. This is consistent with previous reports using murine models, where tissue macrophage numbers were shown to change following chemical exposure (49, 50). Notably, this is the first report of changes in the abundance of CD8^+^ T lymphocytes after xenobiotic exposure. Our understanding of leukocyte subsets in chickens, and reagents to examine them, continues to expand. It will be interesting to take advantage of these in the coming years as we look to gain added resolution into the impact of xenobiotics on subset composition for the leukocyte pool under basal and immune challenged conditions.

**Figure 5:**
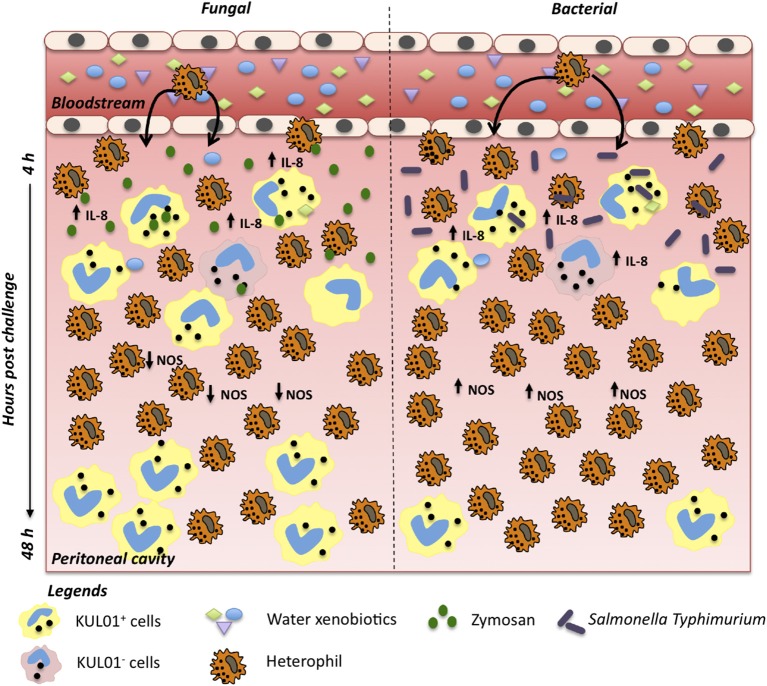
Differential effect of raw water in chicken acute inflammation against fungi and bacterial challenges. After intra-abdominal challenge, following acute exposure to raw water, promotes differential effect on inflammatory process. In a fungi model, we observed marked heterophil infiltration a few hours after *in vivo* challenge, likely driven by a more prominent resident macrophage pool along with an upregulated IL-8, TNF-α, and IL-1β expression. At 12 h post challenge, NO levels and iNOS gene expression were downregulated. Acute inflammatory process is largely complete by 48 h post challenge, allowing a return to homeostasis. In a bacterial model, we also observed marked heterophil infiltration which paralleled up-regulation of IL-8 gene expression. No down-regulation effect was observed in the cellular and molecular levels of NO at 12 h post-challenge. However, 48 h post challenge, heterophil proportions remained higher, while monocyte/macrophage pool remained lower, consistent with a longer acute pro-inflammatory response.

The contributions of immune defenses to host integrity are tightly linked to the effective induction and resolution of inflammatory processes (51–53). Deviations in efficient leukocyte recruitment to infection sites can severely impact host health, disease transmission and performance (52, 53). Our results showed extended heterophil retention within the abdominal cavity for birds in the raw well water group following *in vivo* bacterial challenge (Figure 5). These higher heterophil levels in late phases of acute inflammation coupled to maintenance of ROS and NO production capacity, suggests an extended pro-inflammatory phenotype following bacterial challenge. The concurrent lower proportion of monocyte/macrophages late in the acute inflammation process further suggests a lower capacity and/or slower transition toward activation of tissue repair mechanisms at the infection site. This has broad implications for resolution of inflammatory responses, energy expenditure and the efficient activation of downstream adaptive mechanisms of immunity upon bacterial infection. Interestingly, differential effects were detected in leukocyte recruitment and retention between the fungal and bacterial *in vivo* challenges used in this study. Characterization of phagocyte ROS and NO production also highlighted differential effects among fungal and bacterial challenges. Among the potential implications of these effects, is a differential impact of xenobiotics to the susceptibility of these animals to various infectious challenges.

The impact of raw well water on leukocyte recruitment and function highlighted above paralleled changes observed at the molecular level. The kinetics and absolute levels of gene expression changed for multiple pro-inflammatory mediators, particularly among zymosan challenged birds. Importantly, gene expression of the anti-inflammatory cytokine TGF-β was also upregulated early in the acute inflammatory response among leukocytes derived from the raw well water group. This is consistent with molecular changes observed in other models with individual chemicals, where exposure was shown to promote higher expression in genes including IL-1β, TGF β, and others (50, 54, 55). Together, this suggests dysregulation of the acute inflammatory response following exposure to xenobiotics in drinking water. It will be important to determine if these alterations further compromise the engagement of adaptive mechanisms of immunity and potentially impact the development of long-term protection against pathogens.

Altogether, this work provides added depth in our understanding of the impact of drinking water quality on immune function. Among the greatest advantages of this strategy, is the temporal integration of individual and interactive effects of exposure to multiple contaminants into a few measurable parameters. Our work also demonstrates that these functional platforms can be setup in non-classical animal models to target discrete effects on animal populations that sit at the interface between animal, public and environmental health. As others have already noted, unfortunately, current maximum acceptable levels for individual contaminants in water do not account for the synergistic biological effects of related chemicals and their breakdown products (3, 4, 56, 57). Further, comprehensive screens for individual contaminants are cost prohibitive and thus can only focus on a discrete number of representative compounds. Thus, it is critical that combinatorial effects of chemicals that act through the same or parallel pathways complement existing assessments of water quality. Where possible, performance metrics should also be used as added relevant indicators for the impact of water quality on bird health.

## Data Availability Statement

The raw data supporting the conclusions of this article will be made available by the authors, without undue reservation, to any qualified researcher.

## Ethics Statement

The animal study was reviewed and approved by the University of Alberta Animal Care and Use Committee.

## Author Contributions

JM-B and DB jointly conceived the experimental design. JM-B conducted experiments, analyzed, and interpreted the data. DT conducted qPCR experiments and edited parts of the manuscript. CT contributed with part of *ex vivo* experiments. JW edited part of the manuscript and provided logistic support. JM-B and DB wrote the manuscript. All authors approved this final version.

### Conflict of Interest

The authors declare that the research was conducted in the absence of any commercial or financial relationships that could be construed as a potential conflict of interest.
